# Supplementary figures and images for: Critical role of Babesia bovis spherical body protein 3 in ridge formation on infected red blood cells
Source: PLoS Pathog. 2024 Nov 11;20(11):e1012294. doi: 10.1371/journal.ppat.1012294 (PMC11581398; doi:10.1371/journal.ppat.1012294)

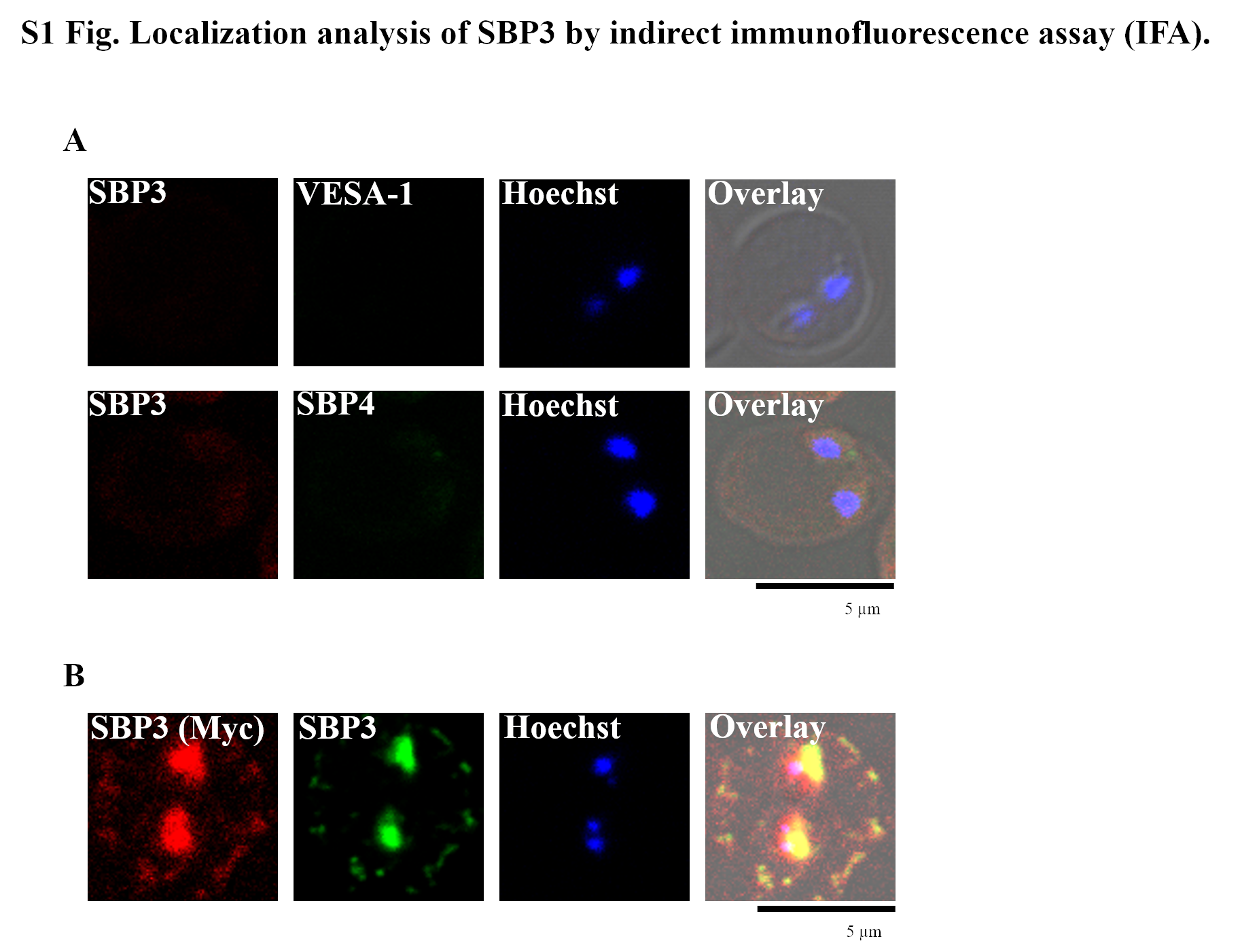

Supplement: S1 Fig — (A) Negative control images of B. bovis SBP3-Myc parasites. The parasites were reacted only with secondary antibodies. Nuclei were stained with Hoechst 33342 (Hoechst, blue). Overlay shows bright field and fluorescent images. Scale bar = 5 μm. (B) Confirmation of stainability of anti-Myc antibody and anti-SBP3 peptide antibody by IFA. (TIF) [file ppat.1012294.s001.tif]

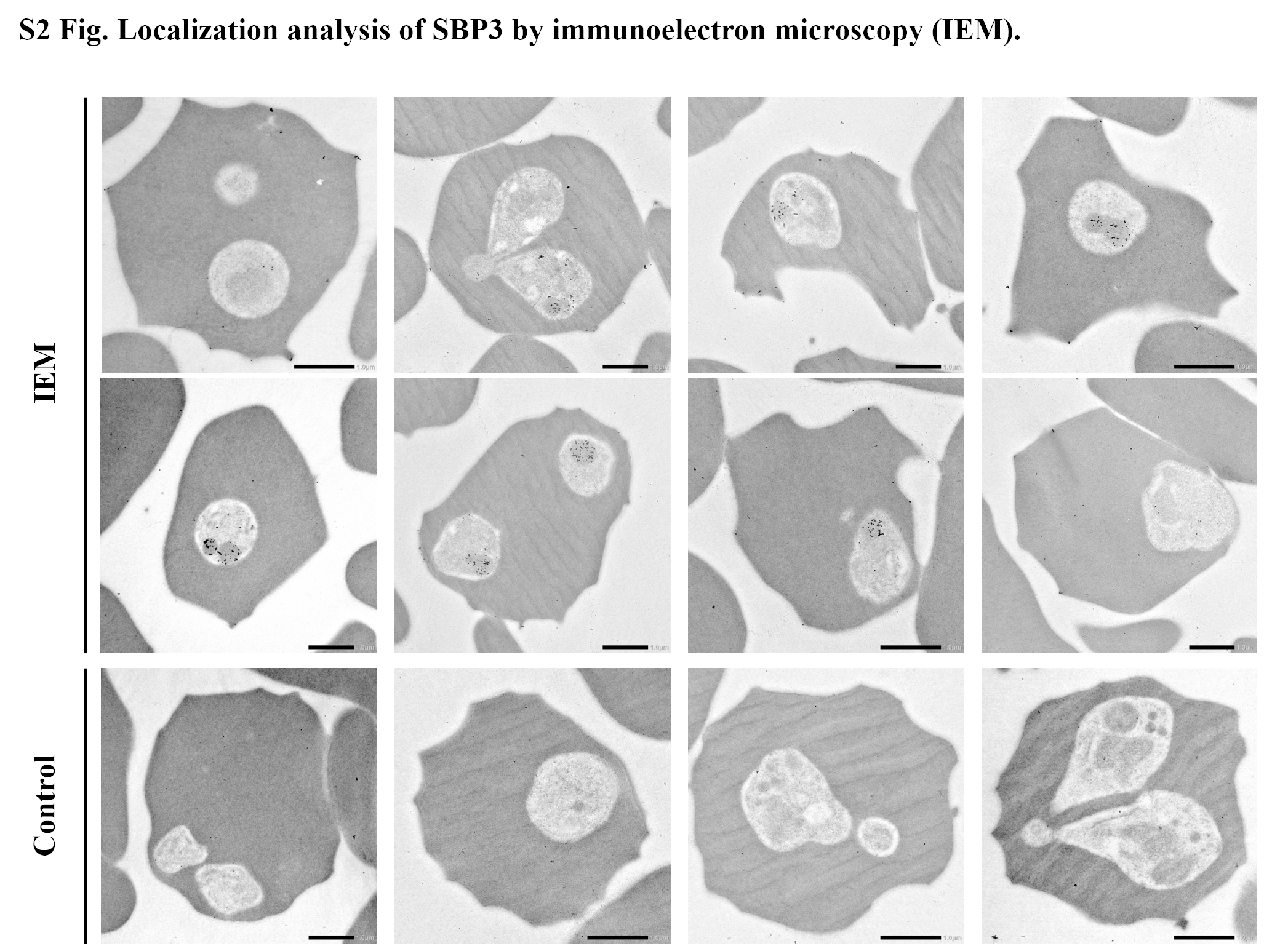

Supplement: S2 Fig — IEM images of B. bovis SBP3-Myc parasites. The parasites were reacted with anti-Myc antibody. Control: negative control images without reaction with anti-Myc antibody. Scale bar = 500 nm. (TIF) [file ppat.1012294.s002.tif]

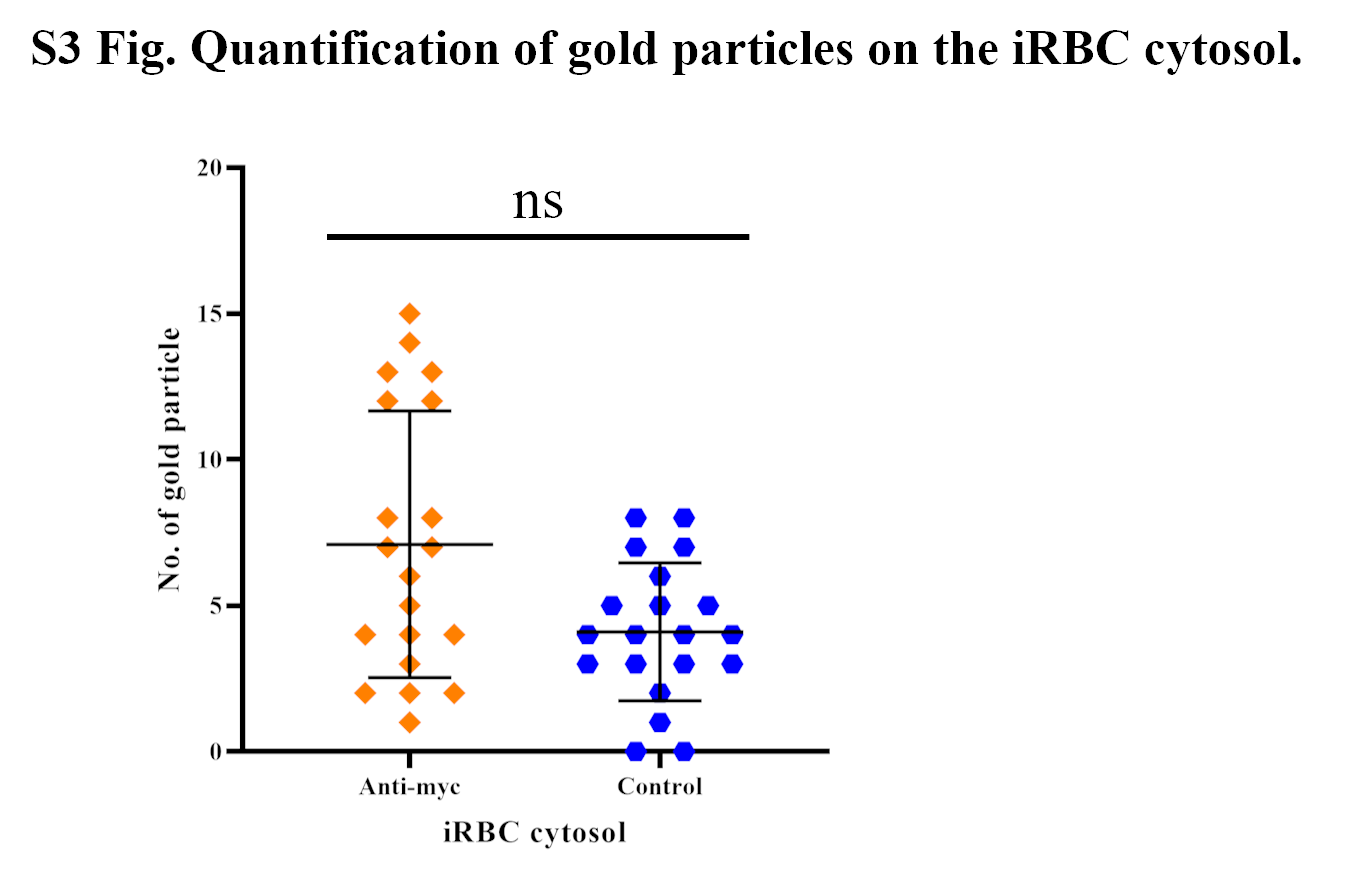

Supplement: S3 Fig — The gold particles were counted for 20 individual images. Anti-Myc: the parasites were reacted with anti-Myc antibody. Control: negative control images without reacting with anti-Myc antibody. The number were 7.1±1.02 and 4.1±0.53 (average ± SE; ns, non-significant; determined by Mann-Whitney U test). (TIF) [file ppat.1012294.s003.tif]

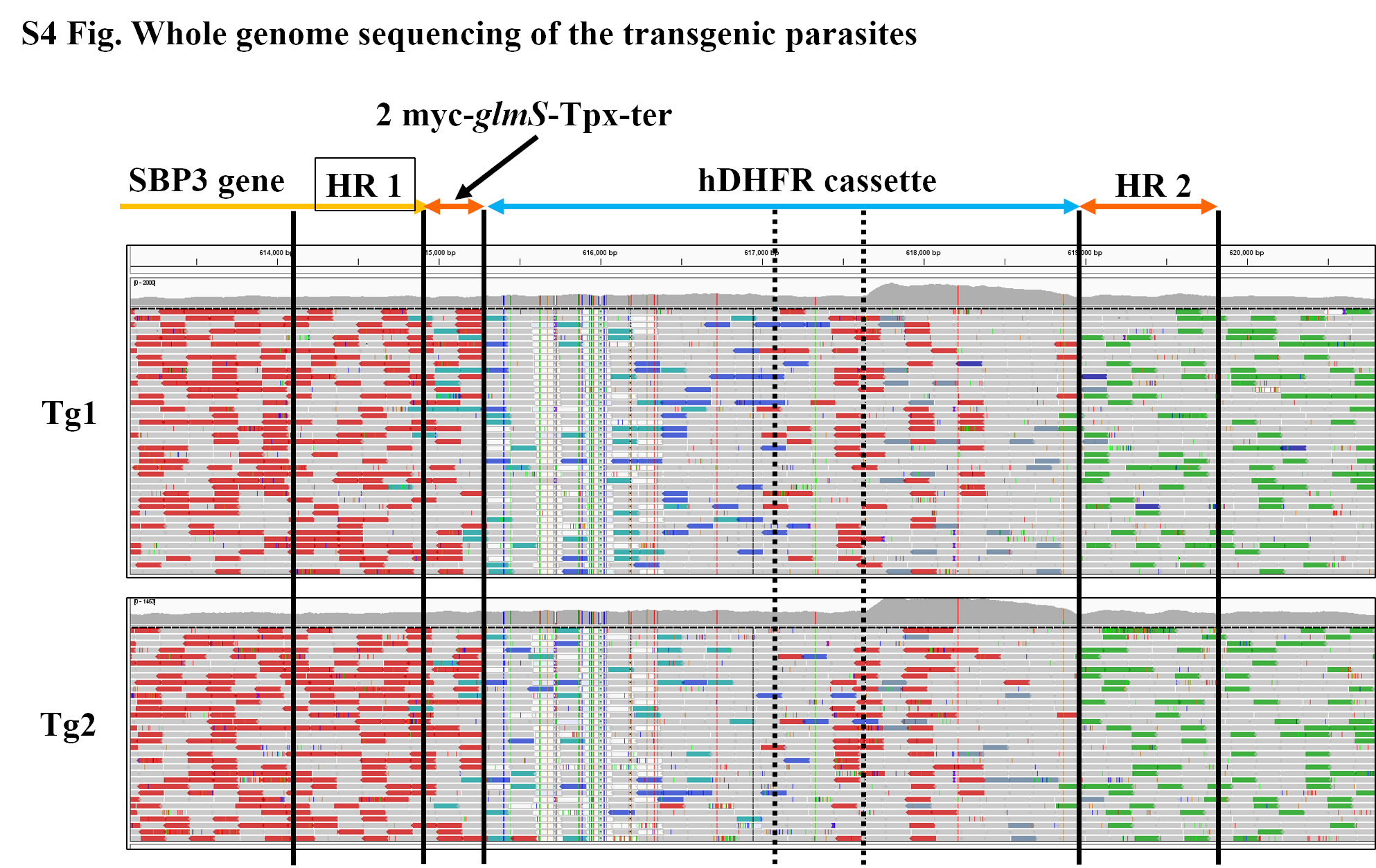

Supplement: S4 Fig — The whole genome of the two clones (Tg1, Tg2) were sequenced by Illumina sequencer. The obtained reads were aligned with the expected recombinant genome sequence, then their sequence depth was examined. (TIF) [file ppat.1012294.s004.tif]

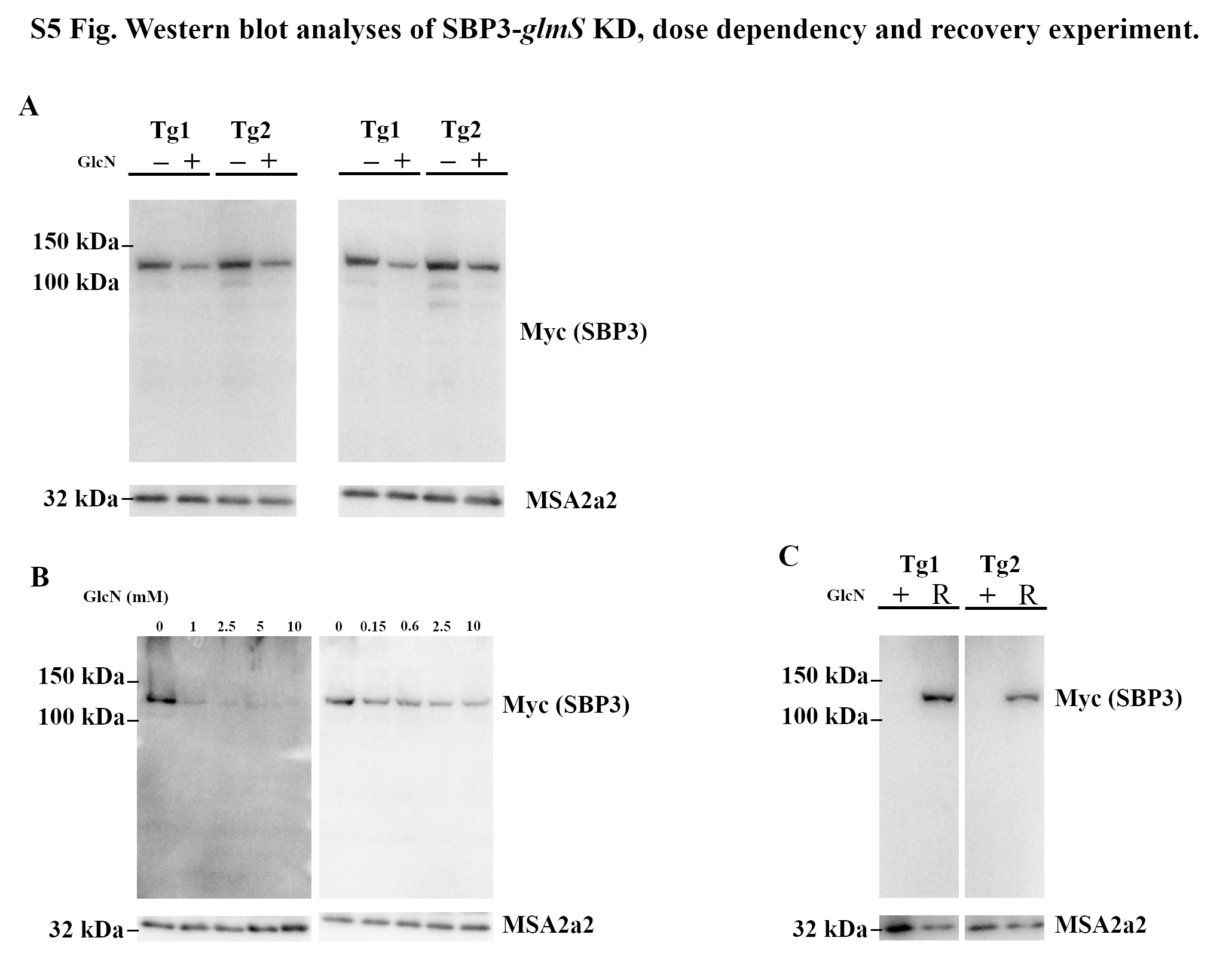

Supplement: S5 Fig — (A) Analysis of SBP3-glmS clones with (+) or without (-) glucosamine (GlcN), Anti-MSA2a antibody was used to detect MSA2a protein as a loading control. The images are other two independent experiments. (B) Western blot analysis of dose dependent GlcN administration on SBP3-glmS parasite. Two experiments were performed administrating 0, 1, 2.5, 5 and 10, or 0, 0.15, 0.6, 2.5 and 10 mM GlcN. (C) KD recovery experiment of SBP3-glmS parasite. +: SBP3 KD with 10 mM GlcN, R: recovery of SBP3 expression after removing the GlcN. (TIF) [file ppat.1012294.s005.tif]

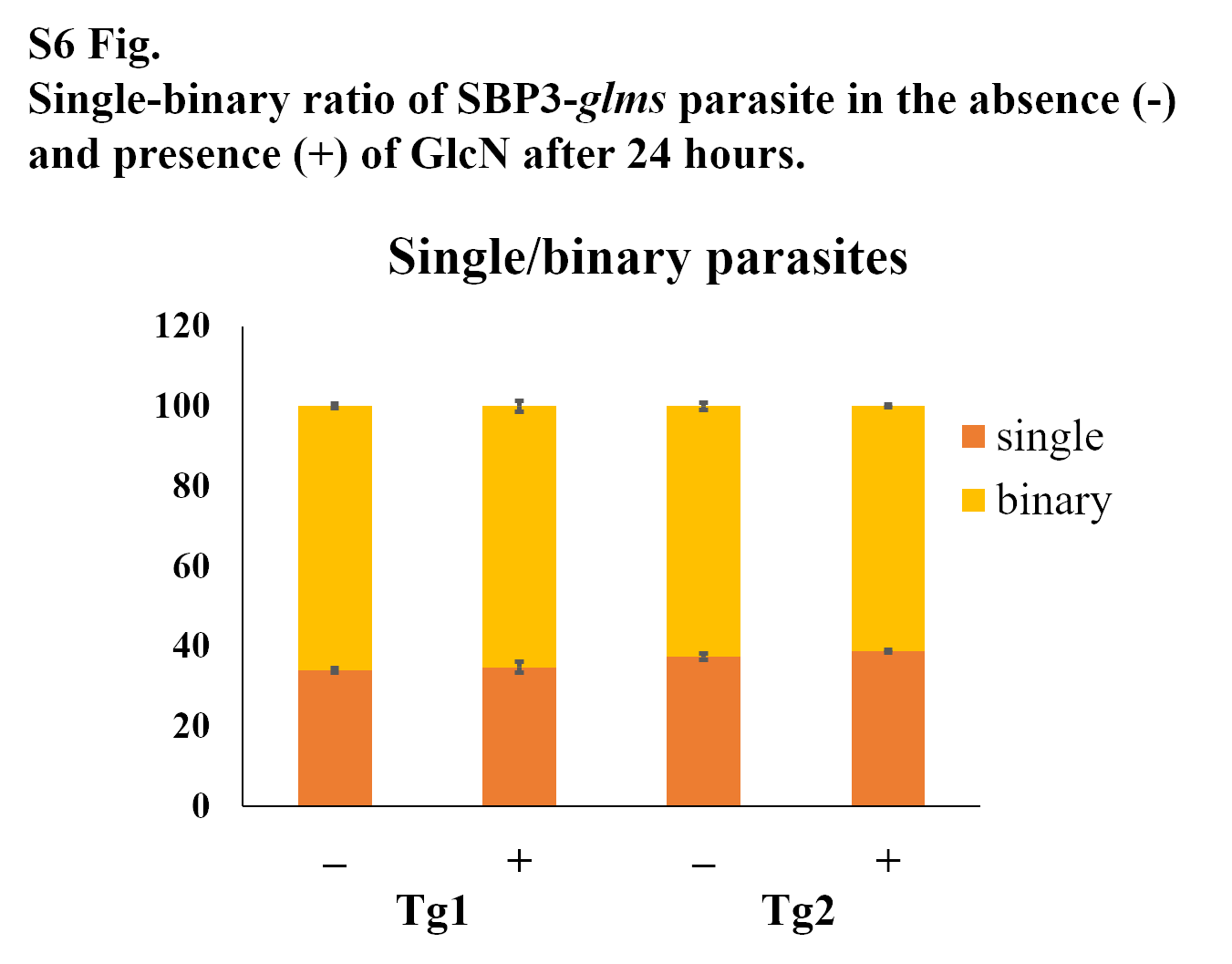

Supplement: S6 Fig — The data shows 3 replicates of two clones, 100 iRBCs were counted (Average±SE). Tg1 showed 34.0±0.5 single/ 66.0±0.5 binary parasites before KD and 34.7±1.3 single/ 65.3±1.3 binary parasites after KD. Tg2 showed 37.3±0.8 single/ 62.7±0.8 binary parasits before KD and 38.7±0.3 single/ 61.3±0.3 binary parasites after KD. (TIF) [file ppat.1012294.s006.tif]

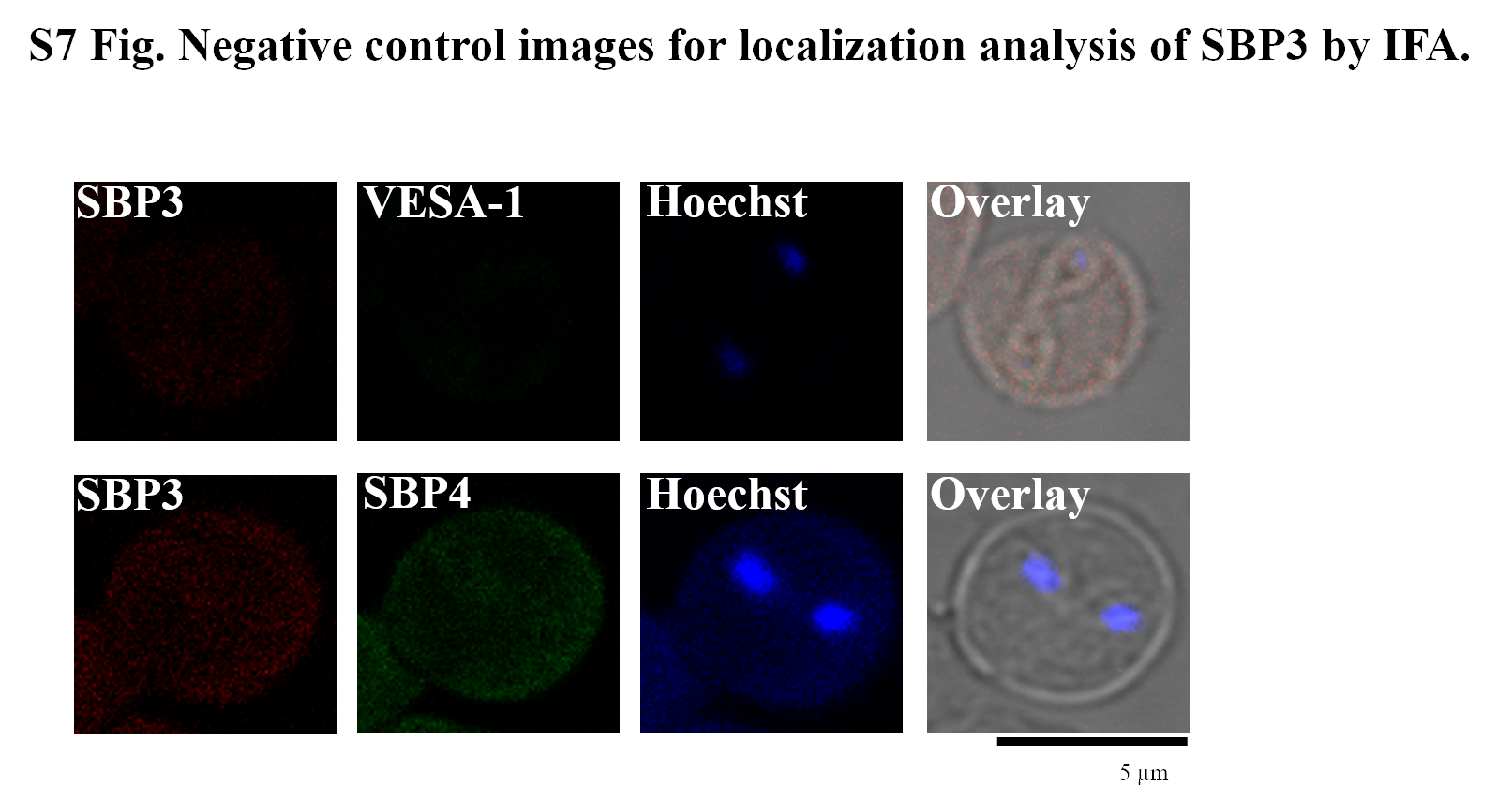

Supplement: S7 Fig — IFA images of B. bovis SBP3-glms parasites. The parasites were stained only with secondary anti-bodies. Nuclei were stained with Hoechst 33342 (Hoechst, blue). Overlay shows bright field and fluorescent images. Scale bar = 5 μm. (TIF) [file ppat.1012294.s007.tif]

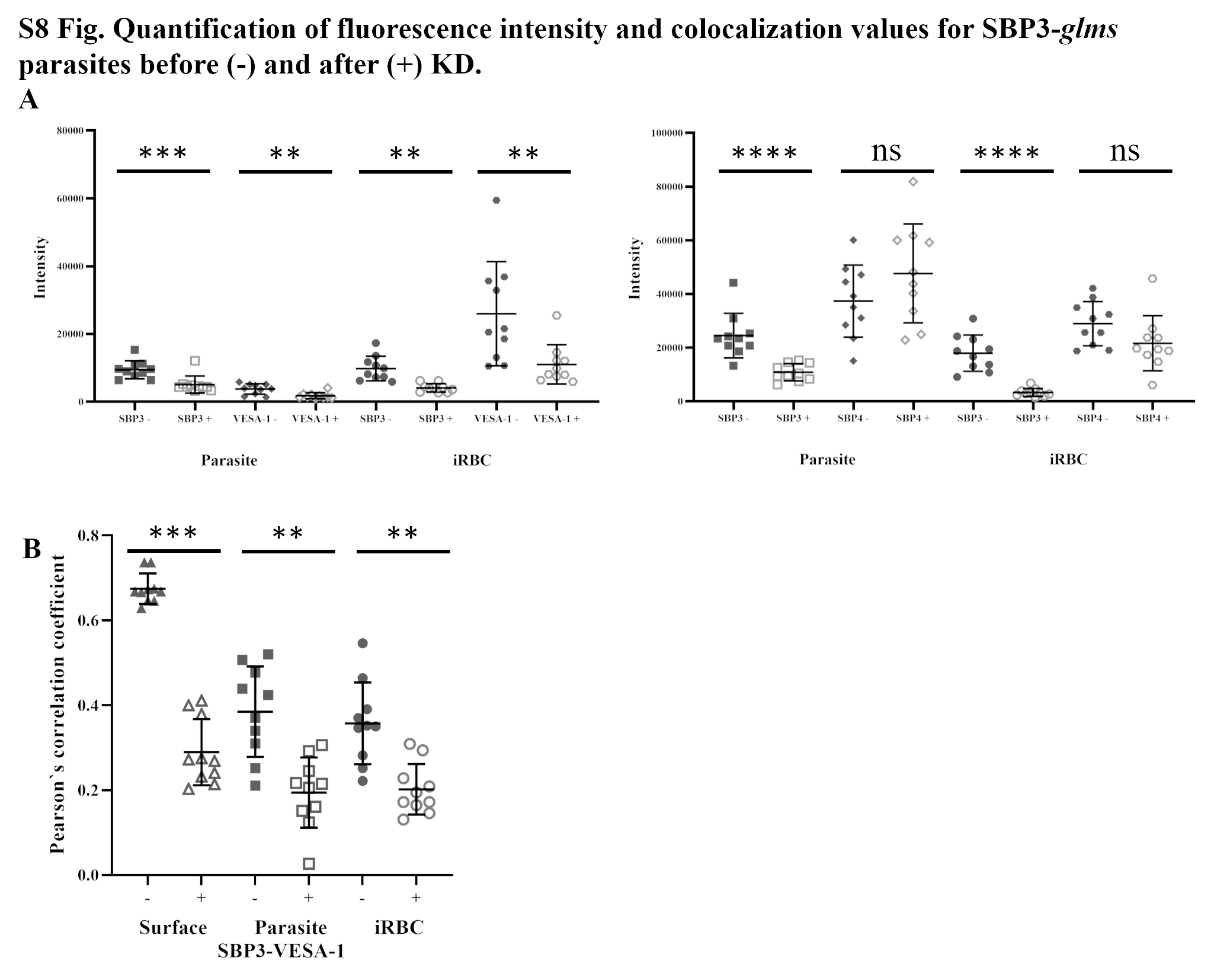

Supplement: S8 Fig — (A) Fluorescence intensity of VESA-1 and SBP4 before and after SBP3 KD on the parasite and cytosol of iRBC for 10 individual images (ns, non-significant; **** p < 0.0001; determined by multiple t-test). (B) Quantification of colocalization between SBP3 and VESA-1 calculated with Pearson’s correlation coefficients for 10 individual IFA images on SBP3-glms parasites before (-) and after (+) KD (** p < 0.01;**** p < 0.0001; determined by multiple t-test). (TIF) [file ppat.1012294.s008.tif]

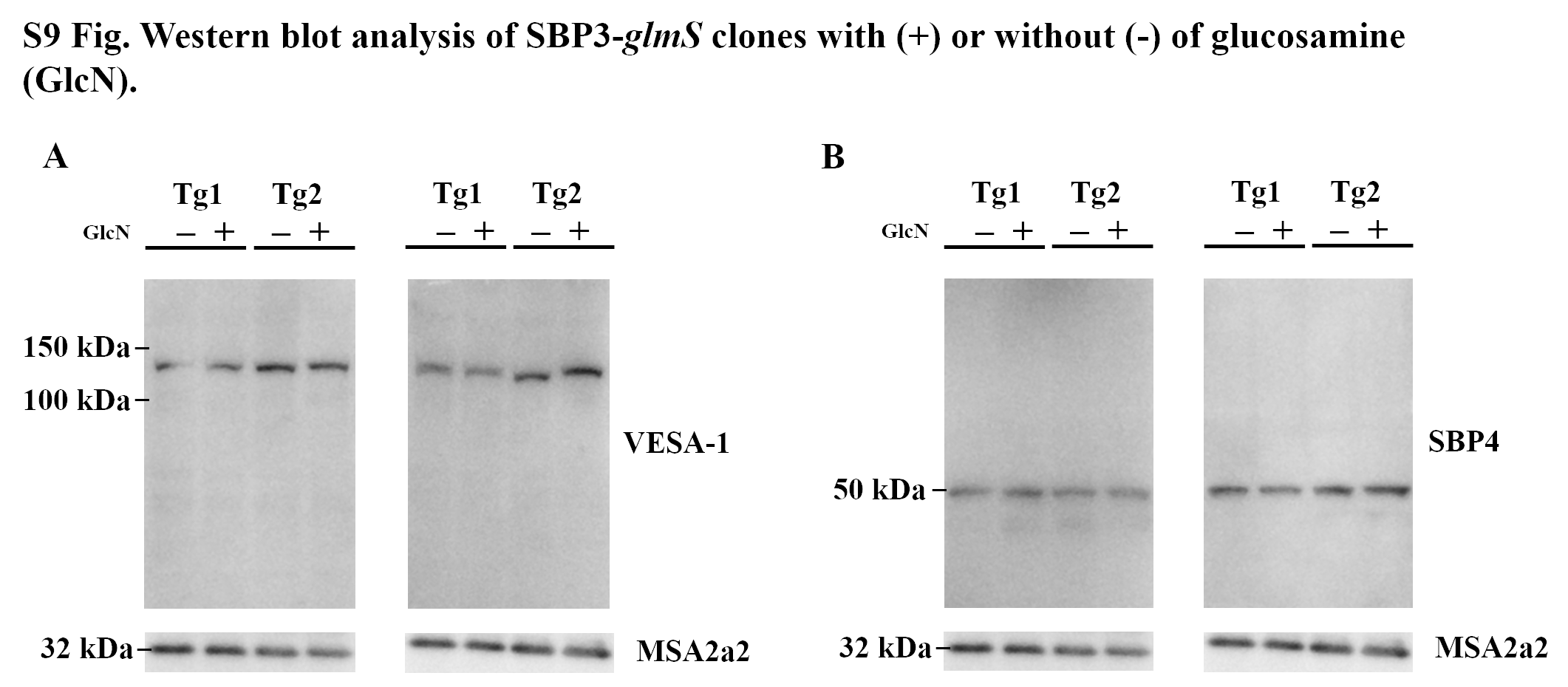

Supplement: S9 Fig — (A) Western blot analysis of SBP3-glmS parasites (Tg1, Tg2) with (+) or without (-) of glucosamine (GlcN). VESA-1: protein was detected by anti-VESA-1 antibody. Anti-MSA2a antibody was used to detect MSA2a protein as a loading control. The image is other two independent experiments. (B) SBP4: protein was detected by anti-SBP4 antibody. Anti-MSA2a: loading control. The image is other two independent experiments. (TIF) [file ppat.1012294.s009.tif]

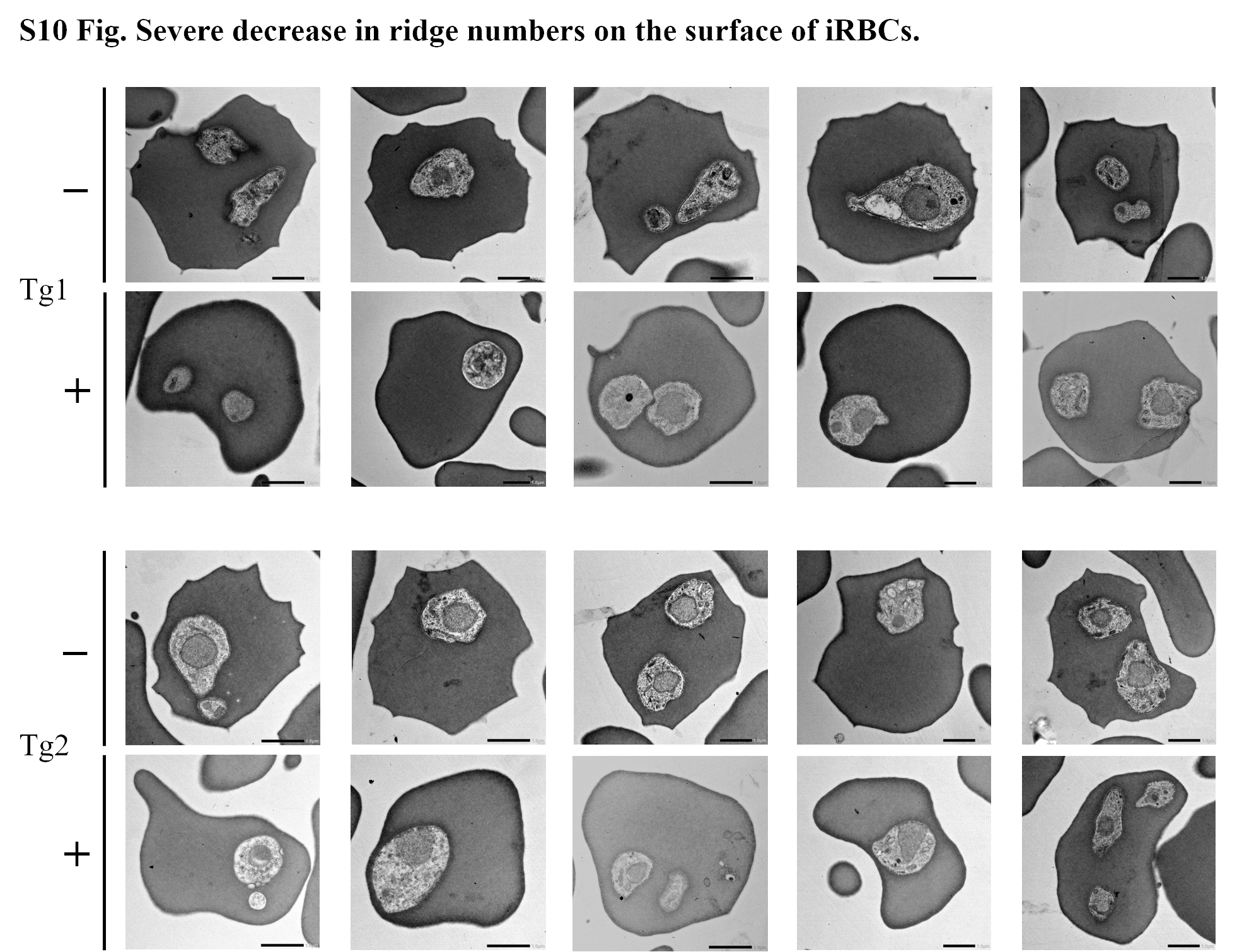

Supplement: S10 Fig — Transmission electron microscopy (TEM) images of SBP3-glmS parasite iRBCs before (-) and after (+) KD for two clones (Tg1 and Tg2). Scale bar = 1 μm. (TIF) [file ppat.1012294.s010.tif]

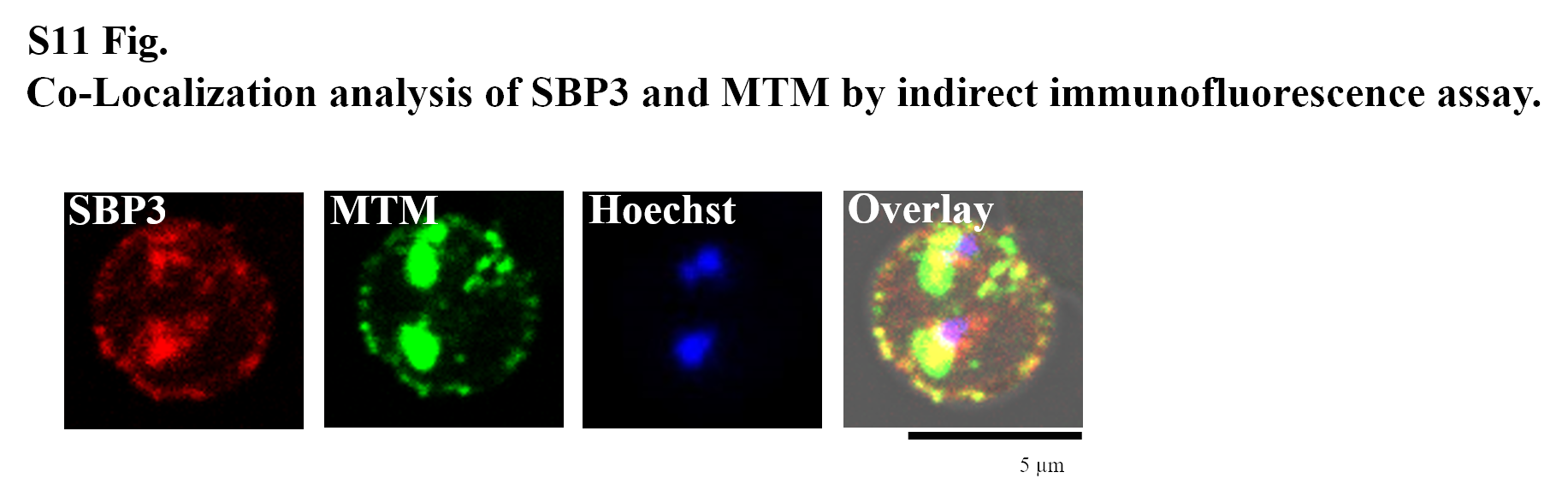

Supplement: S11 Fig — IFA images using B. bovis MTM-Myc parasites [20]. The parasite was stained with anti-SBP3 peptide antibody (SBP3, Red) and anti-Myc antibody (MTM, Green). Nuclei were stained with Hoechst 33342 (Hoechst, blue). Overlay shows bright field and fluorescent images. Scale bar = 5 μm. (TIF) [file ppat.1012294.s011.tif]

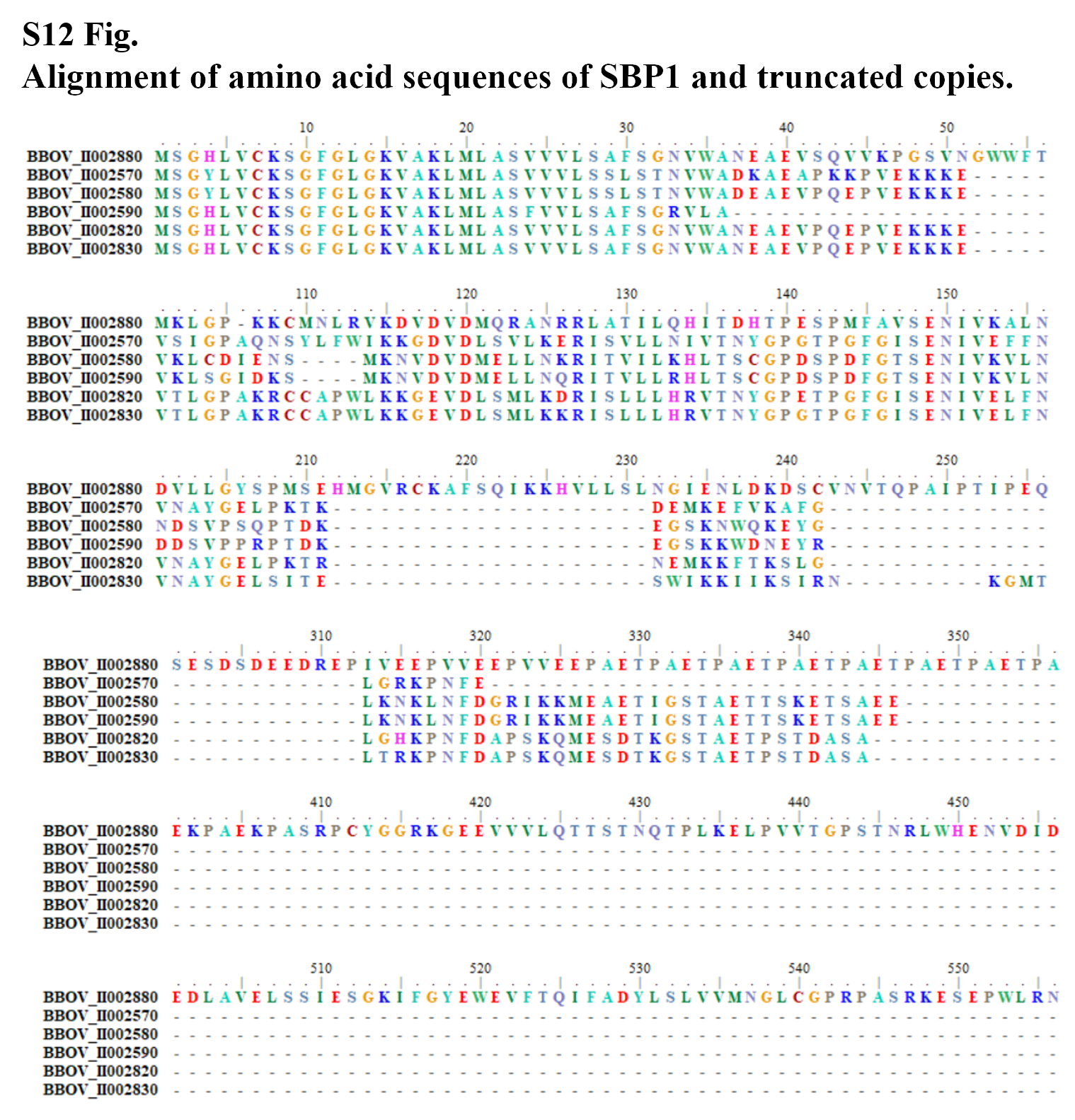

Supplement: S12 Fig — (TIF) [file ppat.1012294.s012.tif]

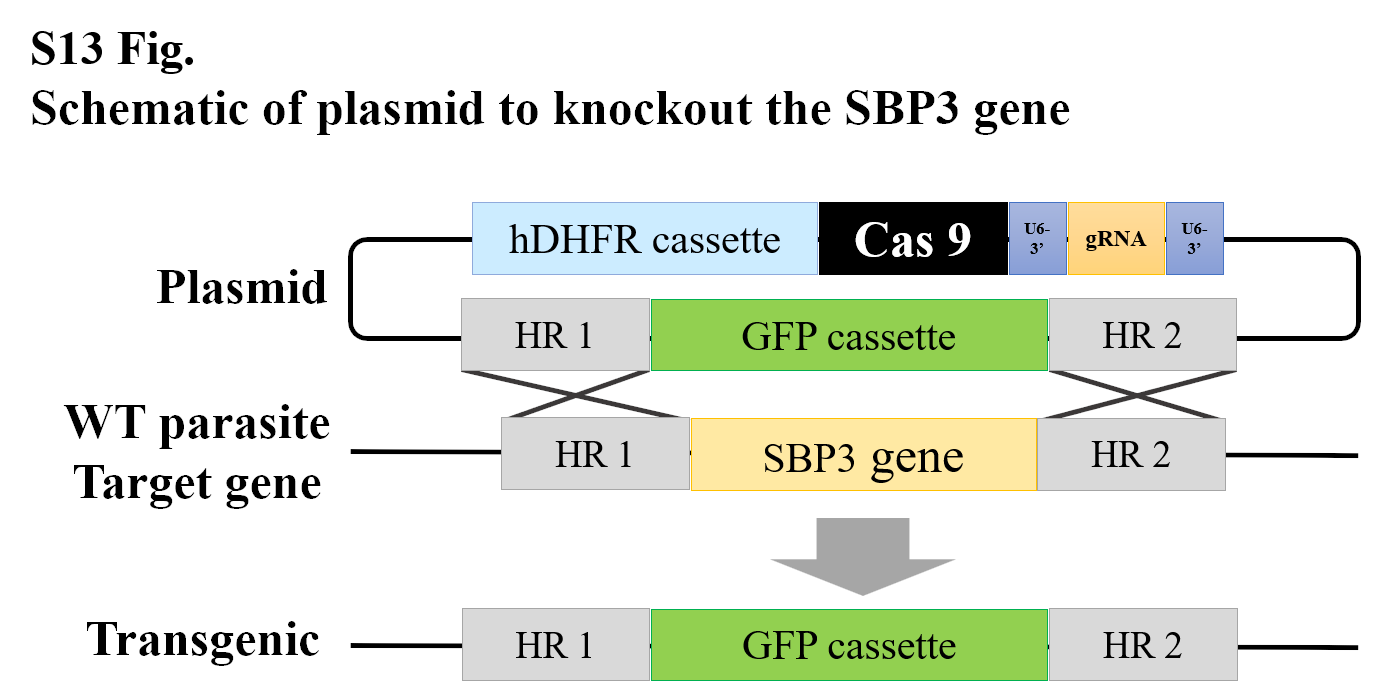

Supplement: S13 Fig — The plasmid was constructed based on Hakimi et al., 2019 [19]. The primers to amplify HR1, GFP cassette, and HR2, and insert guide RNA were shown on S4 Table. (TIF) [file ppat.1012294.s013.tif]

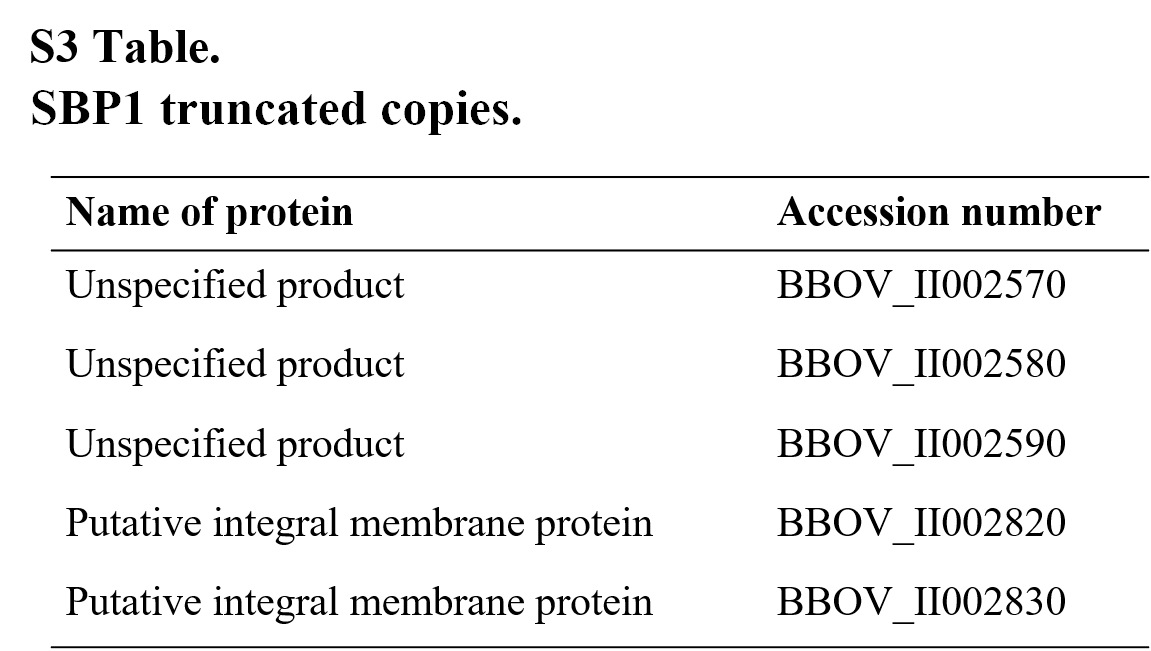

Supplement: S3 Table — (TIF) [file ppat.1012294.s016.tif]

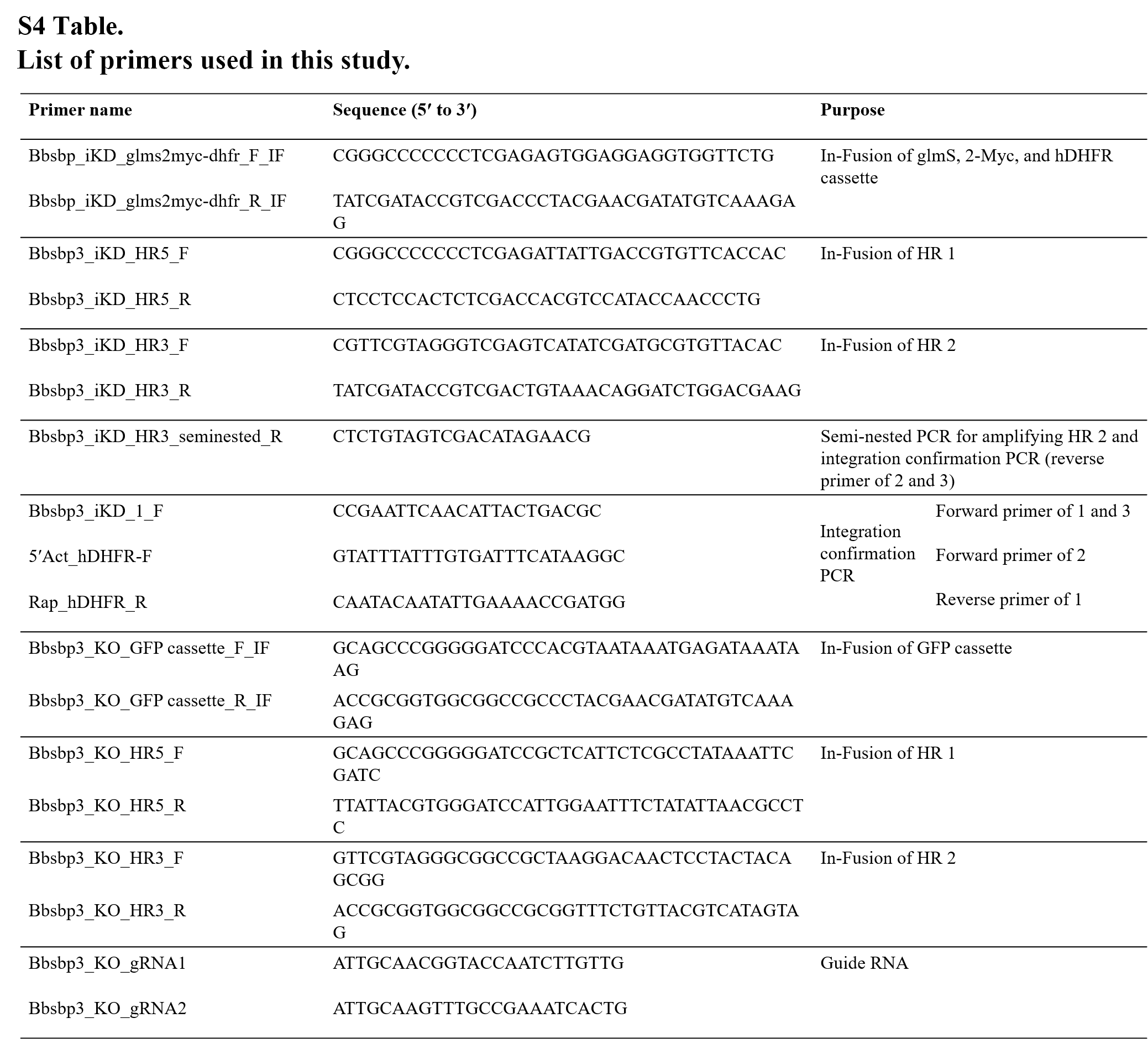

Supplement: S4 Table — (TIF) [file ppat.1012294.s017.tif]
