# Supplementary material for: Critical role of Babesia bovis spherical body protein 3 in ridge formation on infected red blood cells
Source: PLoS Pathog. 2024 Nov 11;20(11):e1012294. doi: 10.1371/journal.ppat.1012294 (PMC11581398; doi:10.1371/journal.ppat.1012294)
Supplement: Attachment — Submitted filename: Reply to the Reviewers comments240925.docx [file ppat.1012294.s018.docx]

Fathi et al., PPATHOGENS-D-24-01111

To the reviewers of this manuscript,

We are grateful for your valuable comments. We have revised the manuscript based on your comments. We have added some control for glucosamine administration experiments. To ensure objectivity of the IFA and IEM images, we quantified the data and also showed additional images on the supplemental materials. Detail answers to your comments and revised places are explained in the following.

Reviewer #1:

 [1] To meet modern standards of rigor formal quantification of co-localization needs to be performed on multiple cells to demonstrate statistically that SBP3 and VESA1 co-localize. It is highly subjective and unconvincing from the overlays alone, especially as these are likely best-available images. Statistical quantification of gold particles in different cellular compartments (on a per-area basis) needs to be performed to demonstrate specific association with spherical bodies. In addition to spherical bodies and the IRBC membrane, considerable gold is observed over non-membrane-bound compartments, the nucleus, IRBC cytoplasm, etc. Also, why is gold found on the external surface of the IRBC membrane (lower panel), too far away to be accounted for by antibody length? Again, distributions of gold are being presented in a very subjective manner that is only partially consistent with the authors’ interpretation and do not meet modern standards of rigor (but potentially could be made to do so).

Quantification of co-localization:

We have analyzed the co-localization for 20 images and added the data of Pearson's correlation coefficient (PCC) values (Line 126-132, 251-252, 526-531, Fig. 1B, and S8 Fig). As we expected, relatively strong colocalization was detected near the surface of iRBC between SBP3 and VESA-1 (PCC=0.72, Fig1B), however, weak colocalization was detected between SBP3 and SBP4 (PCC=0.34). This iRBC surface colocalization between SBP3 and VESA-1 was also detected on SBP3-glmS parasites (n=10, S8 Fig) and the value decreased by SBP3 KD.

Quantification of gold particles:

Number of gold particles was counted for 20 images and statistically analyzed. Though we detected some signals on the surface of iRBC (non-ridge area), the signals were significantly detected at the ridge area (Line 137-145, Fig1D). In addition, gold particles were counted in different cellular compartments, and most of the signals were detected at spherical bodies (Fig 1E). We also confirmed that only a few signals can be detected from the negative control images. The number of gold particles on the iRBC cytosol was higher than negative control images, but not significant (S3 Fig). To ensure the objectivity of the data, we showed several images in the supplemental materials (S2 Fig).

Signal on the external surface of the IRBC membrane:

We think it is difficult to say the exact localization of SBP3 was the inner surface of iRBC membrane or outer surface from IEM images, since the appearance of gold particles can be changed by the angle of the section.

[2] Figure 2 Panel E lacks a wild-type control to demonstrate lack (or presence) of toxicity from long-term treatment with 10 micromolar glcN, an effect that is obscured by the use of a log10 plot. In this reviewer’s experience this is quite a high concentration and can have serious toxic effects, especially after extended exposure, of which there is a common awareness in the malaria community. Without including this control in these experiments the “essentiality” of SBP3 based upon this knockdown experiment is not convincing.

Thank you for your comment. Non-toxicity of 10 mM GlcN to the *B. bovis* has been reported in our previous study (Hakimi et al. 2020), that is why we did not include the data in the initial manuscript. Based on your suggestion, we performed the experiment with parental strain and the result was added to the Fig 2E.

[3] The authors did not attempt to complement the lack of functional SBP3 by simultaneous expression of an unmodified sbp3 gene, a standard approach in genetics studies. Such over-expression should theoretically rescue the cytoadhesive phenotype in the presence of glcN and perhaps even increase it in the absence of glcN. These deficits are critical to the authors’ conclusions regarding SBP3 function in ridge formation, cytoadhesion, and potential pathology.

We believe complementation is common for gene knockout experiment, but not for knockdown approach. We searched several papers which include gene knockdown experiments, however, we could not find papers performing gene complementation experiment (Gabriela et al. 2022, PLOS pathogens, Jonsdottir et al. 2023, PLOS pathogens, Elsworth et al. 2014, Nature, and Vanaerschot et al. 2020, Cell Chemical Biology). On the other hand, we could see some experiments to warrant the knockdown, such as dose dependency of knockdown and recovery from knockdown by removal of the drug. We confirmed that the SBP3 knockdown was induced in a dose dependent manner, and after the removal of GlcN, SBP3 expression was fully recovered (Line 208-215, 507-511). Combine with the data that GlcN administration to the WT parental strain did not change the cytoadhesion phenotype, we believe reliability of the data has increased on the revised manuscript.

Reviewer #2: 1. In Plasmodium, 2-3 mM glucosamine is usually the maximum concentration used because it is mildly toxic to the parasites. Here 10 mM is used so there should be a control for this against wildtype parasite to check for toxicity in Fig 2E. Parasite growth was also quantified by counting smears and ideally the counters should be blinded to the identity of the parasite treatments. This applies to all counting in Figs 4 and 5.

Thank you for your comment. Similar to the comment of Reviewer #1-[2], non-toxicity of 10 mM GlcN to the *B. bovis* has been reported in our previous study (Hakimi et al. 2020), that is why we did not include the data. Based on your suggestion, we performed the experiment with parental strain and the result was added to the Fig. 2E. The counting of the smears was done by an author who performed the experiment. However, during the process of revision, the slides were blindly checked by another laboratory member and also confirmed by the corresponding author. Regarding ridge number counting, the person who took images and counting was different.

2. One of the most unexpected results was that the Babesia parasite grew poorly after GlcN treatment which contrasts with Plasmodium where cytoadherence proteins are usually non-essential. Was any attempt made to investigate why the SBP3 knockdowns were becoming sick? Does SBP3 bind to any RBC or parasite protein that could explain its essential function?

Thank you for your valuable comment. We are also interested in this result, however, essential protein of *Babesia* have not been reported since functional genomics of this parasite is lagged behind. SBP3 is conserved across genus *Babesia* regardless of the cytoadhesion ability of a given species, so we expect that SBP3 has a conserved function in addition to participate in cytoadhesion. We have detected cytoskeleton proteins of bovine RBC and *Babesia* MTM from immunoprecipitation experiment. MTM was suggested as a transporter of drug and sugar from our former study (Hakimi et al., 2020). Still it is just a speculation, but we think MTM and other hypothetical protein which localize at the ridge may be affected by SBP3 KD and impaired parasite growth.

Reviewer #1: A. Major concerns
1. Figure 1. There are multiple problems with this figure. (i) It is unnecessary (and unnecessarily confusing) to label SBP3 red in one part of the figure and green in the next, especially when mouse anti-Myc was used to label SBP3 and rabbit antibodies for VESA1 and SBP4. Please use consistent pseudocoloring of specific antigens in images. Please label as “SBP3” rather than as “Myc”. (ii) Why were nuclear images not blended with the antigen images in the overlays? This would aid in assessing antigen localizations. (iii) What does “GlcN” refer to in this figure? Please remove. (iv) The brightfield images are too dark to contribute any information; please adjust. (v) To meet modern standards of rigor formal quantification of co-localization needs to be performed on multiple cells to demonstrate statistically that SBP3 and VESA1 co-localize. It is highly subjective and unconvincing from the overlays alone, especially as these are likely best-available images. (vi) Statistical quantification of gold particles in different cellular compartments needs to be performed to demonstrate specific association with spherical bodies. In addition to spherical bodies and the IRBC membrane, considerable gold is observed over non-membrane-bound compartments, the nucleus, IRBC cytoplasm, etc. Also, why is gold found on the external surface of the IRBC membrane (lower panel), too far away to be accounted for by antibody length? Again, distributions of gold are being presented in a very subjective manner that is only partially consistent with the authors’ interpretation and do not meet modern standards of rigor (but potentially could be made to do so). (vii) Since rabbit anti-SBP3 antibodies were available (Figure S1), please demonstrate co-localization of SBP3 and Myc tag, using rabbit anti-SBP3 and mouse anti-Myc (wild-type parasites could provide neg. control for Myc). This would help to validate localization of SBP3 and data obtained with the Myc tag; confirmation should be included in the supplement. (viii) Please include in the supplement images of negative controls for IFA and IEM experiments.

Thank you for your comments to improve the manuscript.

(i) We have changed the pseudocoloring of specific antigens. Now SBP3 is shown in red, and VESA-1 and SBP4 are shown in green (Fig 1A, Fig 3A).

(ii) We have revised the overlay images, and included nucleus signal (Fig 1A, Fig 3A).

(iii) It was a typographical error.

(iv) We have changed the brightness of the images (Fig 1A, Fig 3A).

(v) As we explained in comment [1], we analyzed the Pearson's correlation coefficient on 20 images and included the data on Fig 1B.

(vi) As we explained in comment [1], we have counted the number of gold particle on 20 images and included the data on Fig 1D. The number of gold particle was significantly detected at the ridge area than surface of iRBC except ridge. In addition, gold particles were mainly detected at the spherical bodies of the parasites and only a few particles were detected on the other part of the parasites.

(vii) We performed IFA using anti-Myc and anti-SBP3 antibodies and confirmed similar localization (S1 Fig B).

(viii) Negative control data of IFA was shown on S1A Fig and S6 Fig. Negative control data of IEM was shown on S2 Fig and the number of gold particles was shown on Fig 1D, E, and S3 Fig.

2. Figure 2. (i) While the diagnostic PCR results support the desired outcome, a second method is needed to confirm lack of ectopic recombination elsewhere in the genome that may not be detectable by this method. The second method could be anything from qPCR of gene copies to full genome sequencing, but diagnostic PCR alone only detects the expected outcome and no longer meets standards of rigor. (ii) Please explain what was used in panel D as “control”? If Tg1- and Tg2- were used their values should be 1.0. If values were normalized against MSA2a2 or something else, please elaborate. (iii) Panel E lacks a wild-type control to demonstrate lack (or presence) of toxicity from long-term treatment with 10 micromolar glcN, an effect that is obscured by the use of a log10 plot. In this reviewer’s experience this is quite a high concentration and can have serious toxic effects, especially after extended exposure, of which there is a common awareness in the malaria community. Without this control the “essentiality” of SBP3 based upon this knockdown experiment is not convincing.

(i) We performed whole genome sequencing of the transgenic parasite using Illumina sequencer. The sequence data confirmed glmS sequence and hDHFR cassette were successfully integrated at the desired locus and the plasmid construct is not inserted in any other places in the parasite genome (Line 176-178, 489-497, S4 Fig).

(ii) The band intensities were measured by CS Analyzer software on SBP3 and MSA2a signals. The data was normalized by MSA2a and shown in the figure. The value is simply divided the intensity of SBP3 by that of the MSA2a and not adjusted to one.

(iii) As we explained on comment [2], we included WT control on the revised manuscript (Fig 2E).

3. Table S1. How many biological replicates were included? Statistical analysis is lacking.

We have counted 2 more individual experiments and shown in the supplemental material (S6 Fig).

4. Figure 3. (i) See comments about pseudocoloring, labeling, and co-localization analysis in Figure 1; the same issues exist here. (ii) Before claiming “significant reduction of VESA1 signals” please provide actual quantification of signal rather than single images that may or may not be representative. This is an important claim and needs full substantiation. (iii) Please provide band quantification for SBP4 and VESA1 in supplement. (iv) Why is there an apparent change in the size of VESA1 in the Tg1- sample? In that region of the gel this would correspond to 10-15 kDa and possibly a different antigenic variant altogether. How does this affect interpretation of the data?

(i) We have revised the pseudocoloring and labelling (Fig 3A). Co-localization was also analyzed (S8 Fig).

(ii) We have quantified the fluorescence intensity and statistical analysis was performed (Fig 3A, S8 Fig A). Statistical analysis showed significant reduction of VESA-1 signal at the surface of iRBC.

(iii) Band intensities were quantified and shown on Fig 3D.

(iv) We think probably it is because of distortion of the gel or membrane during the electrophoresis and/or transfer. We showed the results of other repeat experiments in S9 Fig.

5. Figure 5. These data are quite clear-cut as far as they go. However, (i) no wild-type parasites were included as a negative control on knockdown/ loss of cytoadhesiveness. Given the number of genes whose regulation was significantly altered by glcN treatment (including up-regulation of BBOV_II005480, an AP2 domain-containing protein and possible transcription factor), and may be affected even in the absence of a glmS tag, it is an overinterpretation to conclude that SBP3 specifically is important to this process. (ii) The authors did not attempt to complement the lack of functional SBP3 by simultaneous expression of an unmodified sbp3 gene, a standard approach in genetics studies. Such over-expression should theoretically rescue the cytoadhesive phenotype in the presence of glcN and perhaps even increase it in the absence of glcN. These deficits are critical to the authors’ conclusions regarding SBP3 function in ridge formation, cytoadhesion, and potential pathology.

(i) We performed cytoadhesion assay using WT parasites and confirmed that cytoadhesive phenotype was not changed by GlcN administration (Fig 5B).

(ii) As we explained at comment [3], we believe complement experiment is not common approach for knockdown experiment. In addition, theoretically SBP3 overexpression can be performed by the second drug selection marker *bsd*/blasticidin-S. However, we should keep pressure of blasticidin-S for episome overexpression and there is no report of safe genome integration locus such as *Plasmodium* *p230p*. It means additional confirmation or control is necessary for complement study and make it harder to evaluate the result.

Minor issues

6. line 62 (and throughout). “functional ortholog” is an inappropriate and meaningless term. SBP3 and KAHRP are not identical in function, and orthologs are defined by their genetic and evolutionary relationships (Tatusov, R.L. et al. 1997. Science 278: 631), not function specifically. “Functional analogs” or “proteins of analogous function” would be terms much more appropriate to this situation.

The words were changed as “functional analog” (Line 62).

7. lines 79-82. This wording makes no sense. Please rewrite for clarity.

We changed as “Cytoadhesion of *B. bovis*-infected red blood cells (iRBCs) to brain microvascular endothelial cells leads to the sequestration of blood flow in the brain vascular system, causing cerebral symptoms and a high fatality rate in infected animals.” (Line 79-81).

8. line 445. Please provide a reference for the anti-VESA1a peptide antibody that demonstrates its universal reactivity with different variant forms of VESA1a.

This antibody was used first time in Hakimi et al., 2020. Though the universal reactivity of this antibody is unclear, we obtained several clones of *B. bovis* and determined expressing VESA1a sequences from *de novo* assembly of RNA-seq data. The peptide was designed from the conserved sequences of the expressing VESA1a.

9. line 203. Before concluding lack of interaction of SBP4 and VESA1, and different functions for SBP3 and SBP4 did the authors attempt co-localization of SBP4 and VESA1? Currently, this feels like too great an extrapolation of existing co-localization data, particularly with the availability of reagents.

Both our VESA-1 and SBP4 antibodies are rabbit antisera, and it made hard to perform co-staining experiment, however, it is valuable suggestion and we consider for future experiment.

10. Figure 4. These are very nice data, but please describe how you defined/identified individual ridges non-subjectively for quantification.

The ridges were defined based on our experiences and counted the ridges on 25 iRBCs. Acquire of the images and the counting of the ridges were performed by different authors, and the data showed clear reduction of ridge numbers on SBP3 KD parasites. To warrant non-subjectively for quantification, we showed several electron microscopy images on S10 Fig.

11. line 246 (and throughout). It is likely an overstatement that cytoadhesion of IRBCs is “the main pathology leading to cerebral babesiosis”, and until better substantiated this stance should be softened. (See conflicting outcomes in this regard in Sondgeroth, K.S. et al. 2013. Parasites & Vectors 6: 181; Nevils, M.A. et al. 2000. Parasitol. Res. 86: 437; Canto, G.J. et al. 2006. NY Acad. Sci. 108: 397). The genesis and involvement of cytokine dysregulation in this form of pathogenesis has not been well studied in bovine babesiosis but may be quite important.

We have softened the explanation as “Because cytoadhesion of iRBCs to BBECs is one of the main pathologies leading to cerebral babesiosis” (Line 302-303).

12. Table 1. Given that proteins were cross-linked in the approach used (rendering this effectively a proximity-labeling study rather than co-precipitation per se), and the reproducibility difficulties of cross-linking studies, it is important to know the number of biological replicates performed in this experiment. How consistent was the capture pattern? Also, please explain the meaning of the numbers given for each co-captured protein (number of fragments? fmolar levels?).

We have shown single immunoprecipitation data on the initial manuscript, we have data of two more biological replicates. The two experiments data was added to S12 table. In brief, spectrin, band 3, ankyrin, protein 4.1, and protein 4.2 were detected on experiment #2, and band3, ankyrin, spectrin, and band 4.2 were detected on experiment #3. The number is number of fragments, so the data is somehow reflecting the abundance, but not reliable as a quantitative mass spectrometry.

13. line 304. Figure S2 shows an alignment of translated sequences, not 5’ and 3’ untranslated sequences. Please correct.

We changed the explanation as “and they shared 5’ and 3’ sequences with high homologies” (Line 363).

14. line 319. Unless attempted many times, failure to recover genetically modified parasites cannot be taken as evidence of essentiality. Please soften the wording.

We avoided to declare “essential” throughout the manuscript.

15. lines 322-327. The essentiality of SBP3 based upon the KD data is unconvincing for the reasons discussed earlier, even though it may be true. Please soften the wording.

We soften the wording as “may play an important role in parasite survival” (Line 378).

16. lines 329-333. Other than loss of ridges, were any morphologic alterations observed in KD parasites?

As we mentioned on Line 276-277, we couldn’t observe clear morphologic alterations other than loss of ridges.

17. line 339. “Knockdown of both..” is ambiguous. Please be specific which proteins were knocked down, and were they knocked down simultaneously?

The explanation was changed as “Knockdown of VEAP in our previous study and SBP3 in this study” (Line 398).

18. line 396. While it is obvious that SBP3 KD affects ridge development it does not prove a vital role for SBP3 in ridge development. This would require additional experimentation. Rather, the results are consistent with it playing such a role and make it an attractive candidate to play such a role.

We think interaction of SBP3 to the cytoskeleton proteins for formation of ridges will require further elucidation as mentioned on Line 460-462. We have softened the explanation of conclusion as “this study revealed that SBP3 plays an important role in ridge formation on iRBCs” (Line 455-456).

19. line 502. Please define “PI”.

We defined PI as “protease inhibitor cocktail solution (PI, cOmplete Mini; Merck)” (Line 535).

20. line 542. Please describe how “band intensities” were quantified.

The band intensities were measured by CS Analyzer software on SBP3 and MSA2a signals (Line 547-548).

Reviewer #2:

3. Is there some way of quantifying the localisation of SBP3 with VESA and SBP4 perhaps by using Pearson’s coefficients for 20-30 cells? There needs to be some quantification.

Thank you for your comment. Same comment was given by reviewer #1, and we have analyzed the co-localization for 20 images and added the data of Pearson's correlation coefficient (PCC) values (Line 126-132, 251-252, 526-531, Fig. 1B, and S8 Fig).

4. In Fig 2A, I can’t tell which of the integration PCR products corresponds to which gel lanes. The primers pairs need to be indicated on the diagram.

To make it easy to understand, the PCR product number 1, 2, 3 were changed to ①, ②, ③ and colored corresponding to the bars. We attempted to put primer pairs name, but it made hard to read the name, we added more explanations to the primer list (S17 Table).

5. For the cytoadherence analysis following SBP3 knockdown, were the people counting the slides blinded to the identity of the parasite treatments?

As explained to comment 1, the counting of the smears was done by an author who performed the experiment. However, during the process of revision, the slides were blindly checked by another laboratory member and again confirmed by the corresponding author.

6. Line 270. Chaperone.

We corrected as “chaperone” (Line327).
