# Supplementary material for: Critical role of Babesia bovis spherical body protein 3 in ridge formation on infected red blood cells
Source: PLoS Pathog. 2024 Nov 11;20(11):e1012294. doi: 10.1371/journal.ppat.1012294 (PMC11581398; doi:10.1371/journal.ppat.1012294)
Supplement: Attachment — Submitted filename: Reply to the Reviewers comments241015.docx [file ppat.1012294.s019.docx]

Fathi et al., PPATHOGENS-D-24-01111R1

To the reviewers of this manuscript,

We greatly appreciate your suggestions. We revised the manuscript according to the suggestions.

Reviewer #2:

1. The authors have satisfactorily addressed the reviews comments. Additional analyses have been performed with statistical support to back up the authors conclusions.

Thank you for your constructive comments for the revision.

2. Line 162. It was not clear to me how the gold particles were counted. Does n=20 mean that the number of gold particles were counted per ridge for 20 ridges and per spherical bodies for 20 of these, etc? If so, please make this clearer.

The gold particles were counted for 20 iRBCs. To clarify this inquiry, we added the explanation “n: the number of iRBCs used for counting the gold particles” (Line 162).

3. Line 187. For 1D Tg1-, the bar goes up to 0.7. Is this because its density is 0.7 compared to the MSA2a2 band which is one? Please be clearer on how the densities are normalised.

The value was obtained by dividing the intensity of SBP3 by that of the MSA2a and not adjusted to one. For example, raw densitometry data was 32565132, 38718476, and 27196539 for SBP3, and 44250953, 45938363, and 41532626 for MSA2a (3 repeat experiments of Tg1- sample). Then, SBP3/MSA2a values were 0.73, 0.84, 0.65 and shown as 0.74±0.05 (average±SE) on Fig 2D.

4. Line 250, what is anti-BSP4?

It was a typographical error and corrected as SBP4.

5. Line 277. Consider indicating where SB are using arrowheads.

Arrowheads were added to the Fig 4A (Fig 4A and Line 284).

6. Why are BBEC called BBMEC on graph 5B?

It was a notation inconsistency, now it was unified as BBEC.

7. Line 317. What about VESA1 expression?

It was reported that *ves1* genes are frequently changing by genomic recombination (Allred and Al-Khedery, 2006), and *ves1* gene sequences are different between our parental strain and T2Bo strain (Asada et al, ICOPA2018). In this study, we aligned the RNA-seq data to the genome sequence of T2Bo strain. Therefore, we know *ves1* gene is expressing from the RNA-seq data, but we cannot evaluate the expression level of each *ves1* gene correctly due to the difference of *ves1* gene sequences.

8. Line 362, Should S14 be S15? There could be other figure labelling mistakes so check them all.

Thank you for pointing it out. It was a mistake and corrected as S15.

9. Line 460. Is “exportation” a real word? Just say “export”

We changed to export (Line 462).
